# Impact of SARS-CoV-2 on the clinical presentation of juvenile idiopathic inflammatory myopathies

**DOI:** 10.1186/s12969-023-00861-4

**Published:** 2023-08-11

**Authors:** Jessica Perfetto, Donna A. Yoo, Carolina Y. Tamashiro, Megan M. Perron, Natalia Vasquez-Canizares, Dawn M. Wahezi

**Affiliations:** 1 Division of Rheumatology, The Children’s Hospital at Montefiore, 3334 Bainbridge Avenue, Bronx, NY 10467 USA; 2 Albert Einstein College of Medicine, Bronx, NY USA; 3 Department of Pediatrics, The Children’s Hospital at Montefiore, Bronx, NY USA; 4 Division of Rheumatology, Children’s Hospital Colorado, Aurora, CO USA

**Keywords:** Juvenile dermatomyositis, Juvenile polymyositis, Juvenile overlap myositis, Juvenile idiopathic inflammatory myopathies, COVID-19, SARS-CoV-2

## Abstract

**Background:**

Growing evidence suggests that infection with severe acute respiratory syndrome coronavirus 2 (SARS-CoV-2) may trigger idiopathic inflammatory myopathies (IIM). Few studies have described individual juvenile IIM (JIIM) cases following SARS-CoV-2 infection, and none explored its potential effects on JIIM clinical presentation. We aim to investigate the impact of SARS-CoV-2 on JIIM in patients diagnosed before and after the onset of the Coronavirus Disease 2019 (COVID-19) pandemic.

**Methods:**

Patients diagnosed with JIIM before age 19 at The Children’s Hospital at Montefiore were included. Demographics, clinical and laboratory data, and evidence of SARS-CoV-2 exposure were collected retrospectively. Patients were grouped by pre-COVID-19 (before January 1, 2020) and post-COVID-19 (January 1, 2020, or later). Descriptive statistics were used to summarize each variable. Non-parametric testing was performed using Fischer’s exact test and Mann-Whitney U test.

**Results:**

Fifty-one patients were included, 13 (25%) diagnosed in the post-COVID-19 era. Of these, 10 (77%) had onset of JIIM symptoms after January 1, 2020; 6 (60%) with known or suspected SARS-CoV-2 exposure. Though not statistically significant, post-pandemic patients tended to be older, female, and have non-specific cutaneous manifestations. Despite reported delays in care for other pediatric diagnoses during the pandemic, fewer post-pandemic patients had delays in JIIM diagnosis.

**Conclusions:**

This is the first study to explore the effects of SARS-CoV-2 on JIIM clinical presentation. While our exploratory single-center study did not find significant differences in JIIM diagnosed pre- and post-pandemic, larger prospective multicenter studies are warranted to evaluate this association and to explore clinical variances over time.

## Background

The link between infectious organisms and autoimmunity has been well-described [1–3]. Given that viruses can induce autoimmunity through various pathways [1], there has been interest since the beginning of the Coronavirus Disease 2019 (COVID-19) pandemic in understanding if and how infection with severe acute respiratory syndrome coronavirus 2 (SARS-CoV-2) impacts onset, flares, and manifestations of autoimmune diseases. SARS-CoV-2 has been shown to activate various parts of the immune system and induce autoimmune diseases [4]. Similarly, viruses may trigger idiopathic inflammatory myopathies (IIMs) [2–4], a group of inflammatory diseases affecting the muscles that can also cause systemic manifestations, including characteristic cutaneous lesions, interstitial lung disease, and myocarditis [4]. IIMs include adult- and pediatric-onset dermatomyositis, polymyositis, and overlap myositis [4]. Immune pathways that become activated and dysregulated in IIMs are also critical in host response against viral infections, including interferon (IFN) pathways [2, 3]. Furthermore, myxovirus resistance protein A, a type I IFN-inducible protein expressed in response to viral infections, has been found in muscle fibers and capillaries in patients with dermatomyositis preceding the development of characteristic perifascicular atrophy [3].

Since the onset of the COVID-19 pandemic, there have been observations worldwide of new diagnosis or flare of existing IIM after SARS-CoV-2 infection and reports of increased IIM incidence [1, 4]. In the pediatric literature, several case reports or small case series described children with newly onset juvenile dermatomyositis (JDM) or flare of existing JDM during the COVID-19 pandemic [3, 5–9], though only a minority demonstrated evidence of preceding SARS-CoV-2 infection [5, 8]. Several noted increased incidence of JDM compared to pre-COVID-19 pandemic [3, 7, 9]. Furthermore, there may be differences in the characteristics of patients diagnosed with JDM during the COVID-19 pandemic, with one study noting older mean age at diagnosis and higher proportion of females compared to patients diagnosed during the 5 years preceding the pandemic [3]. However, the statistical significance of this comparison was not reported.

Although these observational reports identify possible differences in IIM since the COVID-19 pandemic, much remains unknown. While a few reports identify a clear temporal link with evidence of SARS-CoV-2 infection preceding development or flare of IIM [5, 8], the majority either do not describe any exposure to or testing for SARS-CoV-2 [1, 3, 4, 7] or the patients had negative testing [6, 9], limiting the ability to explore the association of SARS-CoV-2 with IIM. Additionally, reports on IIM during the pandemic have been limited to dermatomyositis and JDM; none have directly compared patients with IIM before and after the pandemic [1, 3–9]; and several have reported new diagnoses in insufficient detail to clearly satisfy diagnostic or classification criteria [4]. Understanding the evolving epidemiologic connection between COVID-19 and IIM warrants further exploration to distinguish if cases represent true IIM or rather prolonged post-viral myositis [3]. Furthermore, exploring whether IIM has different clinical manifestations or outcomes since the pandemic also warrants further investigation. In this study, we aim to investigate the impact of SARS-CoV-2 on juvenile idiopathic inflammatory myopathies (JIIM) by comparing baseline clinical manifestations and serologic features in patients diagnosed before and after onset of the COVID-19 pandemic.

## Methods

### Patients

A search of The Children’s Hospital at Montefiore (CHAM) electronic medical record, Epic, was performed using ATLAS, a web-based open-source software application that supports clinical analyses. The search identified JIIM patients seen by a pediatric rheumatologist at CHAM between January 1, 2008 and July 15, 2022. The CHAM catchment and referral area is broad and includes New York City and its suburbs (including Westchester County), New York State, and, less commonly, surrounding states.

JIIM was defined as systemic autoimmune disease characterized by weakness and objective evidence of chronic inflammation of skeletal muscles with onset of symptoms before 19 years of age, based on Rider et al’s definition [10]. Objective evidence of chronic skeletal muscle inflammation included abnormal muscle enzymes or abnormal magnetic resonance imaging (MRI) findings; neither muscle biopsies nor electromyography are routinely performed at our hospital for JIIM. Diseases meeting JIIM definition included JDM, juvenile polymyositis (JPM), and overlap myositis. JDM and JPM were defined based on the Bohan and Peter criteria [11]; overlap myositis required patients to meet criteria for JIIM as well as another autoimmune disease [10]. All International Classification of Diseases (ICD)-10 codes that would capture JIIM patients were queried: M33 (dermatopolymyositis), M60 (myositis), G72 (other and unspecified myopathies), M34.82 (systemic sclerosis with myopathy), M35.03 (Sjogren syndrome with myopathy), G73.7 (myopathy in diseases classified elsewhere), M35.1 (MCTD, mixed connective tissue disease), R76.8 (anti-RNP antibodies present). Patients identified with this search were compared to one of the author’s (DMW) JIIM patient cohort and those of all pediatric rheumatology attendings at CHAM; any who were not captured by ICD-10 code-based search were added. Manual review of all identified patients was performed to exclude patients who did not meet JIIM criteria. Patients were excluded if their initial JIIM clinic appointment was missing, as we were interested in examining clinical features at presentation.

### Data collection

In this retrospective cross-sectional study, manual chart review was performed to assess baseline clinical data. Sociodemographic variables, date of diagnosis and symptom onset, laboratory data, and clinical manifestations were recorded. Muscle weakness was determined using the Childhood Myositis Assessment Scale (CMAS) when available, otherwise physical exam was used. Weakness was categorized using CMAS score based on cutoffs determined by CMAS validation studies [12] or by subjective assessment of degree of weakness based on physical exam when CMAS was unavailable. Muscle weakness, cutaneous manifestations, and laboratory data (excluding autoantibodies) were included from the first pediatric rheumatology appointment (baseline visit). All other clinical manifestations and autoantibodies were included from within the first 6 months of diagnosis to allow for the time required to perform diagnostic testing. Delays in diagnosis were considered as occurring at 6 or more months after symptom onset as this has been reported as the mean time between symptom onset and JDM diagnosis [13].

Patients were grouped by date of diagnosis as pre-pandemic (January 1, 2008 to December 31, 2019) or post-pandemic (January 1, 2020 to July 15, 2022); January 1, 2020 was considered the cutoff date since cases of COVID-19 were first reported in multiple states in the United States around this time. Analysis was also performed using date of symptom onset instead of diagnosis. Evidence of exposure to SARS-CoV-2 was recorded. To assess whether exposure to SARS-CoV-2 may impact subsequent JIIM development, exposure prior to reported symptom onset was used. Exposure was considered known if the patient had a positive polymerase chain reaction (PCR) to SARS-CoV-2 and historical if a household member had confirmed COVID-19 infection but the patient was not tested. Albert Einstein College of Medicine Institutional Review Board (IRB) approval was obtained (approval number 2019–10,210) with waiver of informed consent.

### Statistics

Statistical analysis was performed using STATA software, version 17.0. All variables were examined to identify distribution of continuous variables, missing data, outliers, and potential data entry errors. Descriptive statistics were applied to evaluate baseline characteristics, which were summarized as mean and standard deviation for normally distributed continuous variables, median and interquartile range (IQR) for non-normally distributed continuous variables, and frequencies and percentages for categorical variables. To compare JIIM pre- and post-pandemic, bivariate associations were explored. After checking assumptions of all statistical tests, non-parametric testing was performed using the Mann-Whitney U test for continuous independent variables and Fisher’s exact test for categorical independent variables. Two-tailed alpha of 0.05 was used to determine statistical significance.

## Results

**Fig. 1:**
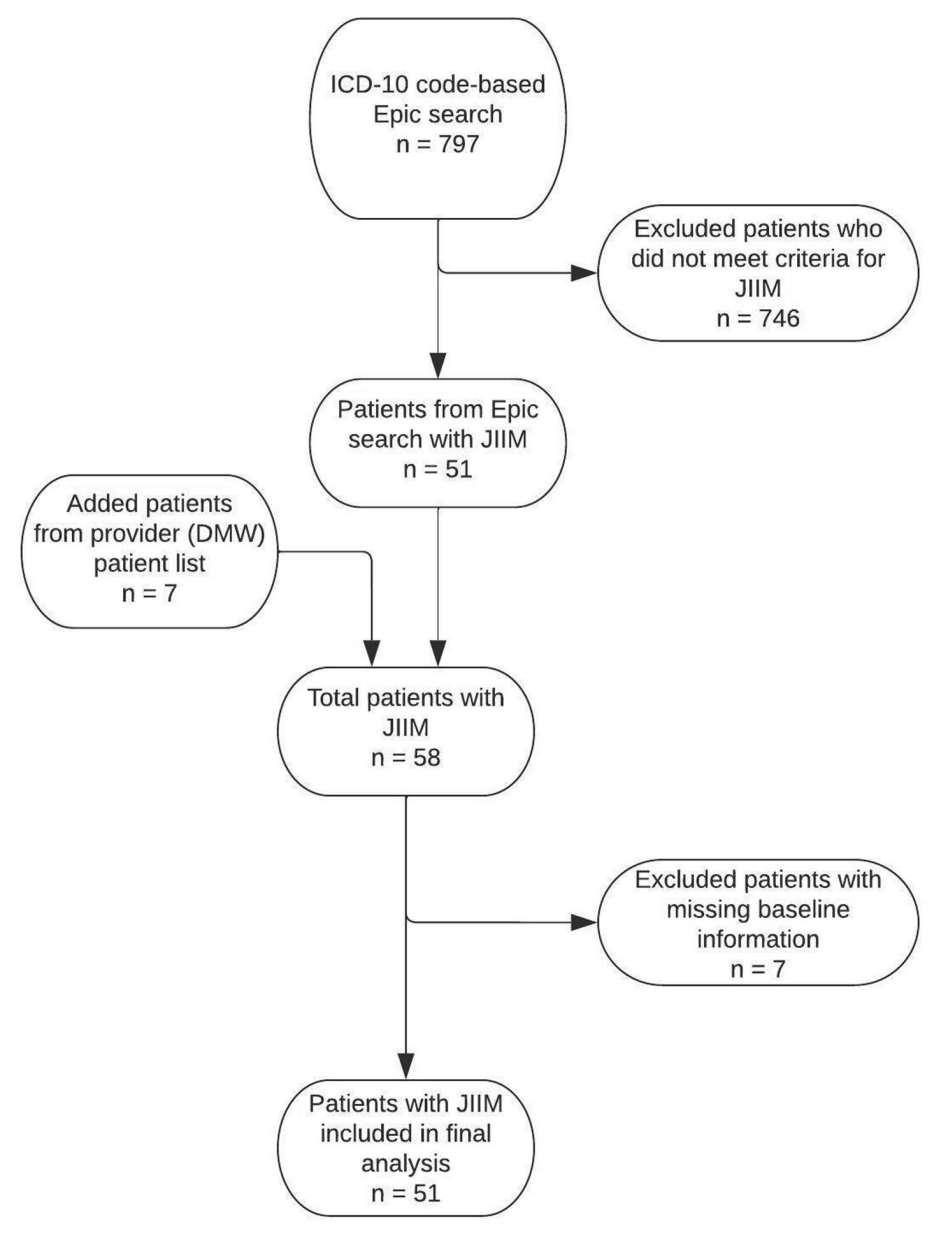
Patient inclusion

The initial ICD-10 code-based search of Epic identified 797 patients. Manual review resulted in removal of 746 patients who did not meet JIIM criteria. Seven patients who were not identified by ATLAS but who were in DMW’s JIIM patient cohort were added to the remaining 51 patients. Of these 58 JIIM patients, 7 patients were excluded due to missing baseline visit information; 51 patients diagnosed with JIIM from January 1, 2008 to July 15, 2022 were included in the final analysis (Fig. 1).

The majority of the 51 JIIM patients had an underlying diagnosis of JDM (n = 43; 84%), were female (n = 42; 82%), and were non-white (n = 41; 80%). The median age at diagnosis was 8.1 years (IQR: 4.1, 12.2). While the median time between symptom onset and diagnosis was 3.5 months (IQR: 2.0, 7.5), 30% of patients experienced delays in diagnosis of 6 months or greater. Most patients had at least 1 autoantibody, with 63% of patients with myositis-specific antibodies (MSA) and 37% with myositis-associated antibodies (MAA) (Table 1).

**Table 1:** Presenting features^a^ in children with juvenile idiopathic inflammatory myopathies (JIIM) diagnosed prior to and after the onset of the COVID-19 pandemic^b^

Variable	Total (n = 51)	Pre-pandemic (n = 38)	Post-pandemic (n = 13)	p-value
Diagnosis				0.52
JDM	43 (84%)	33 (87%)	10 (77%)	
JPM	1 (2%)	1 (3%)	0 (0%)	
Overlap myositis	7 (14%)	4 (11%)	3 (23%)	
Race				0.53
White	10 (20%)	8 (21%)	2 (15%)	
Non-Hispanic Black	14 (27%)	10 (26%)	4 (31%)	
Hispanic	23 (45%)	18 (47%)	5 (38%)	
Asian	3 (6%)	1 (3%)	2 (15%)	
Other	1 (2%)	1 (3%)	0 (0%)	
Sex				0.09
Male	9 (18%)	9 (24%)	0 (0%)	
Female	42 (82%)	29 (76%)	13 (100%)	
Age at symptom onset (years) (n = 43)	8.3 (3.4, 12.8)	7.8 (3.5, 11.5)	8.8 (3.4, 15.1)	0.44
Age at diagnosis (years)	8.1 (4.1, 12.2)	7.4 (4.1, 11.2)	9.3 (5.4, 15.4)	0.17
Time between symptom onset and diagnosis (months) (n = 44)	3.5 (2, 7.5)	4 (2,9)	2 (2,6)	0.41
Delay in diagnosis > 6 months (n = 44)	13 (30%)	11 (35%)	2 (15%)	0.28
Weakness^c^				0.92
None	7 (14%)	5 (13%)	2 (15%)	
Mild	13 (25%)	9 (24%)	4 (31%)	
Mild/moderate	12 (24%)	10 (26%)	2 (15%)	
Moderate	8 (16%)	5 (13%)	3 (23%)	
Moderate/severe	5 (10%)	4 (11%)	1 (8%)	
Severe	6 (12%)	5 (13%)	1 (8%)	
CMAS (n = 33)	39 (27, 46)	39 (25, 46)	39 (37, 45)	0.97
Cutaneous manifestations^d^				0.06
Classic	40 (78%)	31 (82%)	9 (69%)	
Non-specific	4 (8%)	1 (3%)	3 (23%)	
None	7 (14%)	6 (16%)	1 (8%)	
Constitutional symptoms (n = 46)	29 (63%)	20 (61%)	9 (69%)	0.74
Abnormal nailbed capillaries (n = 47)	42 (89%)	31 (89%)	11 (92%)	> 0.999
Cutaneous ulceration (n = 47)	13 (28%)	12 (35%)	1 (8%)	0.08
Raynaud’s (n = 46)	7 (15%)	6 (18%)	1 (8%)	0.65
Calcinosis (n = 50)	10 (20%)	7 (19%)	3 (23%)	0.71
Lipodystrophy	2 (4%)	2 (5%)	0 (0%)	> 0.999
GI involvement	4 (8%)	4 (11%)	0 (0%)	0.56
Cardiac involvement	2 (4%)	2 (5%)	0 (0%)	> 0.999
Pulmonary involvement (n = 50)	6 (12%)	6 (16%)	0 (0%)	0.32
Abnormal muscle enzymes (n = 48)	47 (98%)	35 (100%)	12 (92%)	0.27
Autoantibodies (n = 49)^e^				
None	11 (22%)	7 (19%)	4 (31%)	0.45
Myositis-specific antibodies (MSA)	31 (63%)	24 (67%)	7 (54%)	0.51
Anti-Jo	1 (2%)	0 (0%)	1 (8%)	0.27
Anti-MDA5	9 (18%)	8 (22%)	1 (8%)	0.41
Anti-NXP2	7 (14%)	6 (17%)	1 (8%)	0.66
Anti-Mi2	6 (12%)	4 (67%)	2 (33%)	0.65
Anti-PL-12	1 (2%)	1 (3%)	0 (0%)	> 0.999
Anti-TIF1-γ	10 (20%)	8 (22%)	2 (15%)	0.71
Myositis-associated antibodies (MAA)^f^	18 (37%)	13 (36%)	5 (38%)	> 0.999
Other^g^	7 (14%)	6 (17%)	1 (8%)	0.66

Categorial values reported as n (%) and continuous variables as median (interquartile range). Non-parametric testing performed with Mann-Whitney U Test for continuous variables and Fisher’s exact test for categorical variables

*JDM: juvenile dermatomyositis, JPM: juvenile polymyositis, CMAS: Childhood Myositis Assessment Scale, GI: gastrointestinal*

^a^Presenting features defined as muscle weakness and cutaneous manifestations at diagnostic clinical visit; all other clinical features defined as present within first 6 months of diagnosis

^b^Pre-pandemic defined as diagnosed before January 1, 2020; post-pandemic defined as diagnosed on or after January 1, 2020

^c^Weakness defined by CMAS, with none = 48-52, mild = 45–47, mild/moderate = 39–44, moderate = 30–38, moderate/severe = 16–29, severe = < 15; subjective assessment based on physical exam used when CMAS unavailable

^d^Classic = Gottron’s papules, Gottron’s sign, Heliotrope rash; Non-specific = malar or facial erythema, linear extensor erythema, V sign, Shawl sign, non-sun exposed erythema, extensive cutaneous erythema, livedo reticularis, mucus membrane lesions, Mechanic’s hands, cuticular overgrowth, subcutaneous edema, panniculitis, alopecia

^e^Of the 38 patients with autoantibodies present, 21 had 1 autoantibody, 13 had 2 autoantibodies, 4 had 3 autoantibodies

^f^MAA = anti-RNP, anti-Ro

^g^Other = anti-dsDNA, anti-Smith, anti-rheumatoid factor (RF), anti-cyclic citrullinated peptide (CCP), anti-Scl-70

**Fig. 2:**
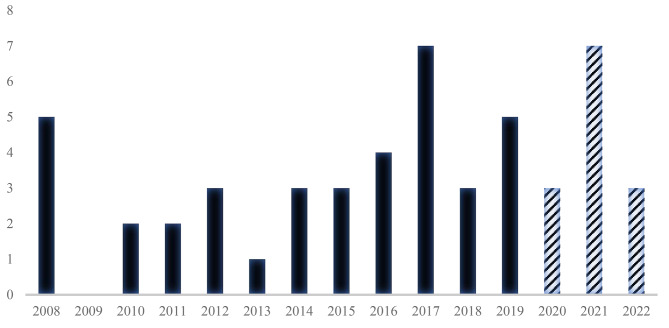
Year of diagnosis (N = 51). Solid bars represent pre-pandemic, patterned bars represent post-pandemic

The distribution of new JIIM diagnoses is depicted in Fig. 2, with peak incidences in our institution in 2017 and again in 2021. Thirteen patients (25%) were diagnosed post-pandemic, of whom 10 (77%) had onset of symptoms after the start of the pandemic (January 1, 2020), whereas the remaining 3 patients had onset of symptoms in 2019. Of the 10 patients, 6 (60%) had known (n = 5, 83%) or historical (n = 1, 17%) exposure to SARS-CoV-2. There was no significant difference in season at the time of diagnosis pre- and post-pandemic (*p* = 0.45).

Overall, there were no statistically significant differences in sociodemographic characteristics, time to diagnosis, or baseline clinical manifestations or laboratory data in patients diagnosed pre- or post-pandemic (Table 1). Most patients, both pre- and post-pandemic, were diagnosed with JDM (87% and 77% respectively, *p* = 0.52) and were non-white (79% and 85% respectively, *p* = 0.53). Compared to patients diagnosed pre-pandemic, those diagnosed post-pandemic did not have significantly different CMAS scores (*p* = 0.97) or degree of weakness (*p* = 0.92), nor was there significantly different absence of autoantibodies (*p* = 0.45) or presence of abnormal muscle enzymes (*p* = 0.27), MSA (*p* = 0.51), or MAA (*p* > 0.999). Though not statistically significant, post-pandemic, patients seemed to have fewer delays in diagnosis (15% versus 35%, *p* = 0.28), were less likely to be male (0% versus 24%, *p* = 0.09), were older at diagnosis (median 9.3 years versus 7.4 years, *p* = 0.17), and had more non-specific cutaneous manifestations (23% versus 3%, *p* = 0.06). None of the patients diagnosed post-pandemic developed lipodystrophy (*p* > 0.999) or had evidence of GI (*p* = 0.56), cardiac (*p* > 0.999), or pulmonary involvement (*p* = 0.32), and only one patient (8%) developed cutaneous ulceration compared to 12 patients (35%) pre-pandemic (*p* = 0.08) (Table 1). Of note, similar results were obtained when defining pre- and post-pandemic by date of symptom onset; however, given date of symptom onset was missing in 8 (16%) patients, date of diagnosis was used in the final analysis.

## Discussion

In our study of 51 children diagnosed with JIIM between 2008 and 2022 at an academic children’s hospital in the Bronx, New York, we did not find any significant differences in baseline clinical manifestations or laboratory features before and after the COVID-19 pandemic. At diagnosis, post-pandemic patients tended to be older and female, paralleling findings from an Iranian study [3]; they also tended to have non-specific cutaneous manifestations and less cutaneous ulceration, though none of these differences were statistically significant. More than half of patients with symptom onset post-pandemic had known or historical exposure to SARS-CoV-2. Only 15% of patients had delays in diagnosis of at least 6 months post-pandemic compared to 35% pre-pandemic. Though not significantly different, this finding is of particular interest given numerous studies worldwide reporting reduced healthcare-seeking behavior with delays in seeking both preventative care and care for a range of diseases, often resulting in more severe disease at presentation [14, 15].

To our knowledge, this is the first study to systematically compare children diagnosed with JIIM before and after the COVID-19 pandemic. There has been suggestion that SARS-CoV-2 may trigger the development of JIIM [3, 5–9] with several reports of increased incidence post-pandemic: an Iranian study noted 8 new cases of JDM from February 2020-February 2021 compared to a usual incidence of 2–4 new JDM cases per year between 2014 and 2019 [3]; a Ukrainian study noted 3 patients diagnosed with JDM in the 11 months between May 2020-April 2021 compared to usual incidence of 1 new JDM patient every 2–3 years [7]; and a Spanish study reported 5 new cases of JDM in the 20 months between March 2020-November 2021 compared to 8 new cases of JDM in the 20-year period from 1999 to 2019 [9]. Others suggest that children with JIIM diagnosed post-pandemic may have different clinical features than those diagnosed pre-pandemic [3]. However, this has been based largely on conclusions drawn from small case series without statistical comparison undertaken. Therefore, our study is important in demonstrating that in our larger cohort of patients, features of JIIM at presentation do not seem to have been significantly impacted by SARS-CoV-2. Additionally, while 2021 experienced the largest number of new JDM patients diagnosed in a single year at our institution after the pandemic, the same number was observed in 2017 prior to the pandemic, suggesting similar incidence patterns pre- and post-pandemic and/or a potential infectious trigger in 2017.

Several studies have identified pathophysiologic links between SARS-CoV-2 and IIMs, including shared immunoglobulin epitope signatures between anti-transcription intermediary factor 1 (TIF1)-γ-positive dermatomyositis patients and SARS-CoV-2 [2] as well as similarities between SARS-CoV-2 infection and positive anti-melanoma differentiation-associated protein 5 (MDA5) dermatomyositis which may be suggestive of a shared pathogenic link [4]. In our cohort, the majority of patients had at least one MSA, with anti-TIF1-γ (p155/140) and/or anti-MDA5 antibodies being the most frequent, which could support the pathophysiologic links described between SARS-CoV-2 and these particular MSAs.

Our study had several notable strengths. It was performed in the Bronx, New York, which has a large Black and Hispanic/Latino population, thus making it generalizable to a broader range of patients from various racial and ethnic backgrounds. Despite the relatively small sample size, to our knowledge it is by far the largest study to evaluate new diagnosis of JIIM since onset of the COVID-19 pandemic. It is also the first study to statistically compare baseline clinical features of JIIM pre- and post-pandemic. We used clear diagnostic, inclusion, and exclusion criteria, in contrast to many other studies exploring the association of SARS-CoV-2 with IIM [4]. Finally, we documented presence or absence of exposure to SARS-CoV-2 in all our patients with symptom onset post-pandemic, with the majority having confirmed or highly suspected infection, in contrast to many prior reports.

Despite these strengths, our study had the limitations intrinsically seen with cross-sectional study designs. This was a single-center study with a relatively small sample size, which likely limited the power to detect statistically significant differences pre- and post-pandemic. Given that this was a single-center study, results may not be generalizable to all centers, despite our relatively racially/ethnically diverse sample. The lack of a control group limited our ability to calculate true incidence rates despite similar incidence patterns pre- and post-pandemic in our study. The ATLAS-based search did not identify 7 JIIM patients; however, we relied on multiple supplementary means of identifying JIIM patients at our center, including querying individual providers and referencing provider JIIM patient lists to minimize the likelihood of missing JIIM patients. Furthermore, due to several patients being diagnosed shortly before data collection and analysis, we limited our comparison to baseline features. Therefore, it remains unknown if manifestations later in disease course, prognosis, and/or outcomes differ in patients diagnosed pre- and post-pandemic. The use of January 1, 2020 as the cutoff date for pre- and post-pandemic may mean that patients without exposure to SARS-CoV-2 were included in the post-pandemic group; while this may have attenuated differences between the groups, this date was chosen to avoid the possibility of inaccurately including patients with exposure in the pre-pandemic group. Finally, although we did have information on exposure to SARS-CoV-2 in all 10 patients with onset of symptoms post-pandemic, not all patients had documented infection. Despite evidence of preceding SARS-CoV-2 infection in 50% and high suspicion of preceding infection in 10% of our post-pandemic patients, we cannot determine if and how SARS-CoV-2 infection impacted the onset or manifestations of subsequent JIIM, nor if this differs from the suspected effects of other viruses, due to the observational nature of the study. Many SARS-CoV-2 infections can be asymptomatic or mildly symptomatic, therefore confirmation of infection can only be obtained with objective testing, which was not possible given the retrospective nature of the study.

## Conclusions

In conclusion, our exploratory study did not find significant differences in JIIM diagnosed pre- and post-pandemic, though non-statistically significant trends emerged. However, larger prospective multicenter studies are warranted to fully evaluate this association and explore whether any differences emerge in disease features or outcomes over time. A larger sample size would also yield greater power to detect differences and allow for better understanding of whether our non-significant but lower rates of delays in diagnosis post-pandemic are significant and, if so, consider why this differs from many pediatric studies demonstrating the opposite. The mechanism by which SARS-CoV-2 impacts JIIM, and if and how this differs from other viruses, also warrants further study.

## Data Availability

The datasets generated and/or analysed during the current study are not publicly available due to HIPAA compliance and this being a single-center study but are available from the corresponding author on reasonable request.

## List of abbreviations

COVID-19: Coronavirus Disease 2019
SARS-CoV-2: severe acute respiratory syndrome coronavirus 2
IIMs: idiopathic inflammatory myopathies
IFN: interferon
JDM: juvenile dermatomyositis
JIIM: juvenile idiopathic inflammatory myopathies
CHAM: The Children’s Hospital at Montefiore
JPM: juvenile polymyositis
ICD: International Classification of Diseases
CMAS: Childhood Myositis Assessment Scale
PCR: polymerase chain reaction
IRB: Institutional Review Board
IQR: interquartile range
MSA: myositis-specific antibodies
MAA: myositis-associated antibodies
TIF1: anti-transcription intermediary factor 1
MDA5: anti-melanoma differentiation-associated protein 5
